# Health professionals’ perceptions about the adoption of existing guidelines for the diagnosis of fetal alcohol spectrum disorders in Australia

**DOI:** 10.1186/1471-2431-12-69

**Published:** 2012-06-14

**Authors:** Rochelle E Watkins, Elizabeth J Elliott, Raewyn C Mutch, Jane Latimer, Amanda Wilkins, Janet M Payne, Heather M Jones, Sue Miers, Elizabeth Peadon, Anne McKenzie, Heather A D’Antoine, Elizabeth Russell, James Fitzpatrick, Colleen M O’Leary, Jane Halliday, Lorian Hayes, Lucinda Burns, Maureen Carter, Carol Bower

**Affiliations:** 1 Telethon Institute for Child Health Research, Centre for Child Health Research, University of Western Australia, Perth, Australia; 2 Discipline of Paediatrics and Child Health, University of Sydney, Sydney, Australia; 3 The Children’s Hospital at Westmead, Sydney, Australia; 4 Department of Health Western Australia, Child and Adolescent Health Service, Perth, Australia; 5 The George Institute for Global Health, Sydney, Australia; 6 National Organisation for Fetal Alcohol Syndrome and Related Disorders, Adelaide, Australia; 7 Menzies School of Health Research, Darwin, Australia; 8 Russell Family Fetal Alcohol Disorders Association, Cairns, Australia; 9 Centre for Population Health Research, Curtin University, Perth, Australia; 10 Public Health Genetics, Genetic Disorders, Murdoch Childrens Research Institute, Melbourne, Australia; 11 Centre for Chronic Disease, School of Medicine, University of Queensland, Brisbane, Australia; 12 National Drug and Alcohol Research Centre, University of New South Wales, Sydney, Australia; 13 Nindilingarri Cultural Health Services, Fitzroy Crossing, Australia

## Abstract

**Background:**

Despite the availability of five guidelines for the diagnosis of fetal alcohol spectrum disorders (FASD), there is no national endorsement for their use in diagnosis in Australia. In this study we aimed to describe health professionals’ perceptions about the adoption of existing guidelines for the diagnosis of FASD in Australia and identify implications for the development of national guidelines.

**Methods:**

We surveyed 130 Australian and 9 international health professionals with expertise or involvement in the screening or diagnosis of FASD. An online questionnaire was used to evaluate participants’ familiarity with and use of five existing diagnostic guidelines for FASD, and to assess their perceptions about the adoption of these guidelines in Australia.

**Results:**

Of the 139 participants surveyed, 84 Australian and 8 international health professionals (66.2%) responded to the questions on existing diagnostic guidelines. Participants most frequently reported using the University of Washington 4-Digit Diagnostic Code (27.2%) and the Canadian guidelines (18.5%) for diagnosis. These two guidelines were also most frequently recommended for adoption in Australia: 32.5% of the 40 participants who were familiar with the University of Washington 4-Digit Diagnostic Code recommended adoption of this guideline in Australia, and 30.8% of the 26 participants who were familiar with the Canadian guidelines recommended adoption of this guideline in Australia. However, for the majority of guidelines examined, most participants were unsure whether they should be adopted in Australia. The adoption of existing guidelines in Australia was perceived to be limited by: their lack of evidence base, including the appropriateness of established reference standards for the Australian population; their complexity; the need for training and support to use the guidelines; and the lack of an interdisciplinary and interagency model to support service delivery in Australia.

**Conclusions:**

Participants indicated some support for the adoption of the University of Washington or Canadian guidelines for FASD diagnosis; however, concerns were raised about the adoption of these diagnostic guidelines in their current form. Australian diagnostic guidelines will require evaluation to establish their validity in the Australian context, and a comprehensive implementation model is needed to facilitate improved diagnostic capacity in Australia.

## Background

Criteria for the diagnosis of fetal alcohol spectrum disorders (FASD) have been available since 1973 and refined periodically [1]. In 1996, the United States Congress charged the Institute of Medicine (IOM) with evaluating existing diagnostic criteria and formulating the best possible diagnostic guidelines reflective of current knowledge [2]. While the IOM guidelines reflected an important advancement in FASD diagnosis, the guidelines remained intentionally broad and conceptual rather than specific and operational [1]. Progress in the field since the publication of the IOM diagnostic criteria has provided the basis for four additional guidelines: the University of Washington (UW) FASD 4-Digit Code in 1997 [3], updated in 2004 [4]; the Centers for Disease Control and Prevention (CDC) FAS guidelines in 2004 [5]; the Hoyme clarification of the IOM diagnostic criteria (Hoyme) in January 2005 [6], and the Canadian FASD guidelines (Canadian) in March 2005 [7]. While areas of debate still exist, these four subsequent guidelines have reached consensus on two fundamental issues: i) that FASD diagnostic evaluation is best conducted by an interdisciplinary team of professionals, and ii) that diagnosis should be based on rigorously case-defined and validated FASD diagnostic guidelines [1]. Despite the availability of FASD diagnostic guidelines for over a decade, many countries, including Australia, have no nationally endorsed guidelines for the diagnosis of FASD.

Reported inconsistencies in the methods used to diagnose FAS in Australia [8,9] and FASD in England [10] suggest that there is limited awareness of appropriate diagnostic guidelines among some clinicians who diagnose FASD. The accurate and reliable diagnosis of FASD presents numerous challenges [11], and opinion is divided on the most appropriate diagnostic methods [1]. In an international survey of diagnostic services for FASD conducted during 2006, only 67.6% of clinics followed a single published diagnostic guideline [12], indicating little agreement on the most appropriate diagnostic criteria. Among the clinics that followed a single published diagnostic guideline, 61% used the UW FASD 4-Digit Diagnostic Code [4] and 35% used Hoyme and colleagues’ [6] revision of the IOM guidelines [2]. Among the clinics that used a combination of guidelines or their own adaption of published criteria for diagnosis, 73% used a combination that included the UW or the IOM guidelines, highlighting their frequent use or adaption in the clinical context.

Until recently there has been no dedicated specialised capacity for FASD diagnosis in Australia, little coordination of service provision, and limited resources to provide the services required by affected individuals and their families. FASD is likely under-diagnosed in Australia, and there is an urgent need to increase diagnostic and management capacity [8,9,13,14]. The adoption of the UW guidelines in Australia has been proposed as an evidence based, sensitive and specific method for the diagnosis of FASD that can account for exposures to other teratogens during pregnancy as well as early life events [9]. Although it is difficult to accurately estimate the prevalence of FASD [10,15], the use of standardised, evidence based diagnostic methods can facilitate meaningful comparison of clinical data [12], provide valuable information about prevalence, and assist planning for the delivery of services for affected individuals [9].

Health professionals’ perceptions about the validity of diagnostic guidelines for FASD, including whether the guidelines produce results that are consistent with their clinical impression, are considered of central importance in determining which diagnostic guidelines for FASD are ultimately used in practice [16]. We require an improved understanding of health professionals’ use of existing guidelines for the diagnosis of FASD in Australia, of factors that influence the use of specific guidelines, and of the suitability of existing guidelines for use in Australia. In this study we aimed to describe health professionals’ perceptions about the adoption of existing guidelines for the diagnosis of FASD in Australia and identify implications for the development of national guidelines.

## Methods

We used an online cross-sectional survey to assess health professionals’ familiarity with and use of existing diagnostic guidelines for FASD, and their perceptions about the adoption of existing diagnostic guidelines in Australia.

### Participant recruitment

We sought to survey a range of health professionals who had expertise in or experience with the screening or diagnosis of FASD, and who were likely to have some knowledge of existing diagnostic methods and guidelines. As health professionals in Australia generally have poor awareness of the diagnostic criteria for FASD [17,18], participants were recruited using three purposive sampling strategies [19] as summarised in Table 1. A formal invitation was sent *via* email to: 57 medical practitioners who had reported a diagnosis of FAS to the Australian Paediatric Surveillance Unit (APSU) surveillance study conducted in 2001–2004 [8]; 128 health professionals who were identified by the study investigators as having experience or expertise in FASD screening or diagnosis, including 14 international experts; and 35 individuals who responded to the canvassing of health professional organisations (including national, state and territory medical and nursing groups) calling for individuals who had relevant experience or expertise.

**Table 1:** Participant recruitment and response to survey questions about existing diagnostic guidelines

**Recruitment category**	**Invited**	**Surveyed**		**Responded**		
	**n**	**n**	**% invited**	**n**	**% invited**	**% surveyed**
APSU^†^	57	40	70.2	20	35.1	50.0
Australian health professionals	114	59	51.8	44	38.6	74.6
Australian professional organizations^‡^	35	31	88.6	20	57.1	64.5
International health professionals	14	9	64.3	8	57.1	88.9
Total	220	139	63.2	92	41.8	66.2

^†^APSU-Australian Paediatric Surveillance Unit.

^‡^ Australian health professional organisations included national, state and territory medical and nursing groups.

Among the 57 medical practitioners who were recruited through the APSU, all were included in the study apart from 17 who either actively declined to participate or who could not be contacted (based on an invalid email address or an automated email reply). Invited health professionals were only surveyed if they actively indicated their willingness to participate in the study. Among the 163 invited health professionals, 64 either did not respond to the email invitation to participate, or declined to participate. The questionnaire was distributed to 139 individuals, including 40 who had reported a diagnosis of FAS to the APSU, 68 who were identified as having experience or expertise in FASD screening or diagnosis (including 9 international experts) and 31 who were recruited from professional organisations.

### Questionnaire

Five existing diagnostic guidelines were identified in a systematic review of the prevention, diagnosis and management of FASD [20]: the IOM diagnostic criteria [2], the UW FASD 4-Digit Diagnostic Code [4], the Hoyme clarification of the Institute of Medicine diagnostic criteria [6], the CDC guidelines for referral and diagnosis of FAS [21], and the Canadian guidelines for diagnosis [7].

Participants were initially asked if they were familiar with each of the five diagnostic guidelines and provided with the response options of ‘yes’ or ‘no’. If participants were familiar with a guideline, three additional questions were asked for each guideline: ‘Have you used this system or guideline?’, ‘Should this system or guideline be adopted as the standard for diagnosis in Australia?’ and ‘Are you aware of, or have you encountered, any limitations of this system or guideline?’ The response options of ‘yes’, ‘unsure’ and ‘no’ were provided for these three questions, and a fourth option of ‘no comment’ was also included for the questions on guideline adoption and limitations to allow participants to indicate that these questions were outside their area of expertise.

If any limitations of a guideline were identified, participants were asked to describe the limitations. Two additional open ended questions sought qualitative information on factors influencing the use of guidelines and perceptions about the adoption of existing guidelines for use in Australia. All participants were asked to ‘Please describe why you use, or don’t use, the diagnostic systems or guidelines listed above’ and to ‘Enter any comments about the adoption of existing FASD diagnostic systems or guidelines for use in Australia’. Demographic characteristics including occupation, country of residence, sex, experience in FASD diagnosis, and completion of training in FASD diagnosis were also assessed.

### Questionnaire administration

The questionnaire on perceptions of existing diagnostic systems was administered as a component of a large survey on the screening and diagnosis of FASD in Australia. The survey was distributed as an online password-protected questionnaire using a secure web server. The questionnaire was delivered to participants using HTML forms which enabled participant responses to be saved to and retrieved from a secure MySQL database. The online questionnaire was pretested by 16 clinicians and health researchers to ensure that the instrument was clear and comprehensible, that the online format functioned on a range of web browsers and operating systems, and that the questionnaire had face validity.

An email containing a personal username, password and a link to the questionnaire website was sent to all invited participants who were asked to complete the questionnaire within 14 days. Email reminders were sent approximately 7 days and 2 days prior to the response deadline. The response deadline was subsequently extended by 8 days to facilitate an improved response, and one attempt to contact non-responders was made by telephone for 43 non-responders for whom we had telephone contact information.

### Analysis

Descriptive statistics were generated for each question using PASW Statistics version 18.0.1 (SPSS Inc., 2009). The chi-square test was used to assess associations between categorical variables, and Fisher’s exact test was used for 2 by 2 tables where any expected cell values were less than 5. All analyses were evaluated with two-tailed test statistics and effect sizes are described using proportions, or risk ratios with 95% confidence intervals (CI). The statistical analyses presented here are exploratory, and no adjustment has been made for multiple comparisons.

Qualitative data were independently coded and analysed by two investigators using qualitative inductive content analysis methods [22,23]. Data from each open ended question were reviewed alongside the quantitative data and coded inductively based on the underlying meaning of the responses. Coding schemes used by both analysts were documented and reviewed for consistency to ensure the credibility and trustworthiness of the analysis process [22]. Approval for this study was granted by The University of Western Australia Human Research Ethics Committee and the Western Australian Aboriginal Health Information and Ethics Committee.

## Results

Of the 139 health professionals surveyed, 92 (66.2%) responded to the questions on existing diagnostic guidelines (Table 1). Participants included 38 paediatricians (41.3%), 23 non-paediatrician medical practitioners (25.0%) and 31 other health professionals (33.7%), including nurses, allied health professionals and clinical researchers. Three quarters of participants were female, and paediatricians were less likely to be female (50.0%) than other participants (92.6%) (*χ*^2^ (1, N = 92) = 22.3, p < 0.001). Participants were predominantly Australian residents (91.3%), and five of the eight international participants were residents of New Zealand. Over a quarter of participants (26.7%) had completed specific training in the diagnosis of FASD and 76.7% reported experience in either the screening or diagnosis of FASD. Paediatricians were more likely to have been involved in diagnosis (75.7%) than other participants (25.9%) (*χ*^2^ (1, N = 90) = 22.3, p < 0.001).

### Familiarity with existing diagnostic guidelines

Among the 92 participants, 38.0% were not familiar with any diagnostic guideline, 21.7% were familiar with 1 guideline, 13.0% were familiar with 2 guidelines and 27.2% were familiar with 3 or more guidelines. Approximately half of participants were familiar with the UW guidelines, and over a third of participants were familiar with the Canadian guidelines (Table 2). Paediatricians were more likely to be familiar with the CDC guidelines than other health professionals (Table 2). Familiarity with one or more guidelines was associated with occupational group, country of residence, experience in diagnosis, and completion of training in diagnosis (Table 3).

**Table 2:** Comparison of familiarity with and use of existing diagnostic guidelines between paediatricians and other occupations

**Guideline**	**Familiar with guideline**			**p**^**‡**^	**Used guideline**^**†**^			
	**n (valid % of Occupation n)**				**n (valid % of Familiar n)**			
	**All**	Paediatrician (n = 37)^*^	Other (n = 54)^*^		**All**	**Paediatrician**	**Other**	**p**^**‡**^
IOM (n = 88)	27 (30.7)	12 (33.3)	15 (28.8)	0.65	8 (29.6)	3 (25.0)	5 (33.3)	0.70
UW (n = 89)	45 (50.6)	21 (58.3)	24 (45.3)	0.23	25 (55.6)	15 (71.4)	10 (41.7)	0.04
CDC (n = 90)	28 (31.1)	16 (43.2)	12 (22.6)	0.04	12 (42.9)	8 (50.0)	4 (33.3)	0.38
Canadian (n = 91)	32 (35.2)	15 (40.5)	17 (31.5)	0.38	17 (53.1)	8 (53.3)	9 (52.9)	0.98
Hoyme (n = 88)	12 (13.6)	6 (16.7)	6 (11.5)	0.54	6 (50.0)	2 (33.3)	4 (66.7)	-

IOM-Institute of Medicine guidelines; UW-University of Washington 4-Digit Diagnostic Code; CDC-Centers for Disease Control and Prevention guidelines; Canadian-Canadian guidelines; Hoyme-Hoyme *et al.* clarification of Institute of Medicine guidelines.

^†^Only participants who were familiar with a guideline were required to respond to this question.

^‡^2-tailed p-value for chi-square (or Fisher’s exact) test comparing the proportion of paediatricians *versus* other occupations. Results not reported for all comparisons due to small numbers.

^*^Denominators vary due to missing data, as summarised in column 1.

**Table 3:** Individual characteristics and familiarity with and use of one or more existing diagnostic guidelines

**Characteristic**	**Familiar with one or more guidelines (n = 92)**			**Used one or more guidelines**^**†**^** (n = 57)**		
	**yes n (%)**	**no n (%)**	**p**	**yes n (%)**	**no n (%)**	**p**
Occupation			0.004			0.26
paediatrician	30 (78.9)	8 (21.1)		23 (76.7)	7 (23.3)	
other	27 (50.0)	27 (50.0)		17 (63.0)	10 (37.0)	
Country of residence			0.02			0.09
Australia	49 (58.3)	35 (41.7)		32 (65.3)	17 (34.7)	
other	8 (100)	0 (0.0)		8 (100)	0 (0.0)	
Experience in diagnosis^‡^			<0.001			0.003
yes	33 (82.5)	7 (17.5)		28 (84.8)	5 (15.2)	
no	23 (46.0)	27 (54.0)		11 (47.8)	12 (52.2)	
Completed training^‡^			<0.001			0.10
yes	22 (91.7)	2 (8.3)		18 (81.8)	4 (18.2)	
no	34 (51.5)	32 (48.5)		21 (61.8)	13 (38.2)	

^†^Participants who were not familiar with a guideline were not required to respond to these questions. Also excludes participants who did not respond to the question or who indicated that the question was outside their area of expertise.

^‡^Due to missing data, actual n is lower than reported in the column heading.

### Use of existing diagnostic guidelines

Among the 57 participants who were familiar with one or more guidelines, 29.8% had not used any guideline, 40.4% had used one guideline, 19.3% had used two guidelines and 10.5% had used three or more guidelines. The UW and Canadian guidelines were the two most frequently used guidelines, and over half of the participants who reported being familiar with these guidelines also reported having used them (Table 2). These findings indicated that as a proportion of all 92 participants, 43.5% reported use of any guideline, 27.2% reported use of the UW guidelines, and 18.5% reported use of the Canadian guidelines. Among participants who were familiar with the UW guidelines, paediatricians were 1.7 (95%CI 1.0-3.0) times more likely to have used the UW guidelines (71.4%) than other health professionals (41.7%, Table 2).

Participants who had experience in diagnosis were 1.8 (95%CI 1.1-2.8) times more likely to report using one or more guidelines (84.8%) compared with those who did not have experience in diagnosis (47.8%, Table 3). Analysis of the association between use of the UW and Canadian guidelines (the two most frequently used guidelines) and country of residence, experience in diagnosis and completion of training found that international participants were 2.1 (95%CI 1.2-3.6) times more likely to have reported using the Canadian guidelines (87.5%) than Australian participants (41.7%) (p = 0.04, Fisher’s exact test). There was only weak evidence of a similar trend for the UW guidelines, with international participants 1.7 (95%CI 1.1-2.7) times more likely to have reported using the UW guidelines (85.7%) than Australian participants (50.0%); (p = 0.11, Fisher’s exact test).

Participants who had experience in diagnosis were 2.9 (95%CI 1.2-7.0) times more likely to have used the UW guidelines (72.4%) than participants who did not have experience in diagnosis (25.0%) (*χ*2 (1, N = 45) = 9.7, p = 0.002). Similarly, participants who had completed training in diagnosis were 2.7 (95%CI 1.5-4.7) times more likely to have used the UW guidelines (88.9%) than participants who had not completed training in diagnosis (33.3%) (*χ*2 (1, N = 45) = 14.9, p < 0.001). There was only weak evidence of a similar trend for the Canadian guidelines, with participants who had experience in diagnosis 1.8 (95%CI 0.8-4.0) times more likely to have used the Canadian guidelines (64.7%) than participants who did not have experience in diagnosis (35.7%) (*χ*2 (1, N = 31) = 2.62, p = 0.11). There was no evidence that participants who had completed training in diagnosis were more likely (risk ratio = 1.1, 95%CI 0.5-2.1) to have used the Canadian guidelines (52.9%) than participants who had not completed training in diagnosis (50.0%) (*χ*2 (1, N = 31) = 0.03, p = 0.87). The power of these analyses is limited by the small number of participants who reported having used the UW or Canadian diagnostic guidelines.

### Adoption of existing diagnostic guidelines

Approximately one third of participants who were familiar with the UW guidelines recommended that they be adopted in Australia, as did 30.8% of participants who were familiar with the Canadian guidelines. In comparison, almost two thirds of participants who were familiar with the Hoyme guideline, and just less than half of participants who were familiar with the IOM guideline, recommended that they not be adopted in Australia (Table 4).

**Table 4:** Health professionals’ perceptions about the adoption of existing diagnostic guidelines

**Guideline**	**Recommend adoption**^**†**^				**Identified limitations**^**†**^			
	**total n**	**yes n (%)**	**unsure n (%)**	**no n (%)**	**total n**	**yes n (%)**	**unsure n (%)**	**no n (%)**
IOM	25	1 (4.0)	13 (52.0)	11 (44.0)	21	7 (33.3)	11 (52.4)	3 (14.3)
UW	40	13 (32.5)	20 (50.0)	7 (17.5)	31	12 (38.7)	13 (41.9)	6 (19.4)
CDC	23	2 (8.7)	13 (56.5)	8 (34.8)	22	6 (27.3)	11 (50.0)	5 (22.7)
Canadian	26	8 (30.8)	14 (53.8)	4 (15.4)	22	8 (36.4)	9 (40.9)	5 (22.7)
Hoyme	11	1 (9.1)	3 (27.3)	7 (63.6)	9	7 (77.8)	1 (11.1)	1 (11.1)

IOM-Institute of Medicine guidelines; UW-University of Washington 4-Digit Diagnostic Code; CDC-Centers for Disease Control and Prevention guidelines; Canadian-Canadian guidelines; Hoyme-Hoyme *et al.* clarification of Institute of Medicine guidelines.

^†^Participants who were not familiar with a guideline were not required to respond to these questions. Also excludes participants who did not respond to the question or who indicated that the question was outside their area of expertise.

The perceived presence or absence of limitations for the five guidelines is summarised in Table 4. The proportion of participants who identified limitations for a specific guideline was highest for the Hoyme guideline. Coding of the qualitative data identified seven categories of perceived limitations that were either not specific to any single guideline, or that were reported for two or more guidelines. These included: i) that existing guidelines had not been demonstrated as relevant for the Australian population, including the lack of Australian normative data, particularly for the assessment of facial characteristics among Indigenous Australians and those from culturally diverse backgrounds; ii) the use of the diagnostic terminology ‘alcohol-related’, which implies that alcohol is ‘the causative factor’ for neurodevelopmental disorder; iii) limitations of methods used to evaluate prenatal alcohol exposure: including that under-diagnosis of neurodevelopmental disorders may occur in cases of suspected but unconfirmed alcohol exposure, or when exposure is unknown, such as in foster children; and conversely that the perception that ‘any’ confirmed alcohol exposure was considered ‘too gross a measure’ of exposure; iv) the use of ‘less conservative case definitions around central nervous system abnormality, facial features and growth impairment’ which lack specificity may lead to over-diagnosis, including use of the 10th percentile criterion for palpebral fissure length and the requirement for only 2 facial features for a diagnosis of FAS; v) a ‘heavy emphasis on dysmorphology assessment’ including the use of physical abnormalities as the sole basis of the FAS diagnostic criteria, and a perceived ‘over emphasis on occipital frontal circumference’; and conversely, that the need for evidence of significant central nervous system dysfunction and a ‘reliance on testing for the 9 central nervous system domains’ might ‘prevent a diagnosis of FAS from being rendered in children under the age of 8 years’ when ‘early accurate diagnosis is paramount to effective intervention’; vi) the lack of use of an interdisciplinary approach to diagnosis, or alternatively, that an interdisciplinary team for diagnosis is ‘not always available’; and vii) a general lack of evidence to support guideline recommendations, including the perception that the evidence for the amalgamation of the components of other guidelines is unclear.

In addition, the following perceived limitations were reported: that the IOM guidelines lack objectivity and rigour, including use of a ‘gestalt approach’ to assess facial features which ‘is not sufficiently case-defined to render accurate and consistent diagnoses under the umbrella of FASD’; that the Hoyme guidelines are ‘the only diagnostic system that maintains the classification of alcohol-related birth defects’ which has been omitted from other diagnostic guidelines; that the UW guidelines are ‘too complex to be useable clinically outside of research’, include ‘too many possible diagnoses’, that training was considered necessary to use these guidelines, and that ‘the language should be modified for clinical utility’; and that the CDC guideline ‘only provides diagnostic criteria for full FAS’.

All participants were also asked why they did or did not use any of the five diagnostic guidelines. Responses from 47 participants (51.1%) indicated that diagnosis was outside their area of expertise, that they only contribute to diagnosis (*i.e.* conduct relevant assessments that inform diagnosis, but do not make the final diagnosis), or that they only refer cases for diagnosis, and 13 participants (14.1%) did not respond to the question. Among the remaining 32 participants, use of diagnostic guidelines was based on: their familiarity with the guideline or completion of training based on a specific guideline (34.4%); relevance and ease of use in the clinical context, including use across the lifespan or in settings where alcohol exposure cannot be confirmed, and the need to consider many different conditions and causes (34.4%); the validated, objective and comprehensive nature of the guideline (18.8%); the use of information or tools from a number of guidelines to inform clinical judgement as the guidelines are ‘not set in concrete’ (25.0%); and problems with the practical application of guidelines in a clinical environment, including complexity, time constraints, and disagreement with the content of guidelines (21.9%).

Comments were received from 26 participants on the adoption of existing FASD diagnostic guidelines in Australia. They indicated: that a standard national approach, based on the adoption and adaption of existing guidelines, and which is valid in the Australian context and appropriate for use in rural and remote areas, is required (26.9%); that providing education, training and support for health professionals who are going to use the diagnostic tool is key (23.1%); that an inclusive, interdisciplinary and inter-agency model of service delivery is required which engages parents and carers and ensures access to diagnostic, intervention and support services (23.1%); that Australian normative data are required, particularly for facial norms among Indigenous Australians (19.2%); that an Australian guideline must be easy to understand and practical to use (11.5%); that specific guidelines should be adapted for use in Australia (15.4%); and that standardised photo analysis methods should be used to supplement clinical examination of facial dysmorphology (7.7%). One participant (3.8%) also recommended the use of newer scientific methods including functional neurological imaging techniques.

## Discussion

Participants were most familiar with, most frequently used, and most frequently supported the adoption of the UW and Canadian guidelines in Australia. However, no single existing guideline received a high level of agreement about its adoption for use in Australia, and participants were most likely to indicate that they were unsure about whether existing guidelines should be adopted. Although participants in our study were recruited based on their expertise with or involvement in the screening and diagnosis of FASD, only 43.5% of participants had used diagnostic guidelines for FASD, and there was general uncertainty about the suitability of existing guidelines for use in Australia. Our findings are consistent with the limited knowledge about FAS among health professionals [18], perceptions that it is more difficult to diagnose FAS among Indigenous Australians [18,24], the lack of national recommendations on diagnosis in Australia, and the limited availability of information on the comparative performance of existing diagnostic guidelines to inform clinical decision making.

When evaluating the suitability of existing diagnostic guidelines for use in Australia, participants recognised a need for guidelines that are evidence based, relevant to the local clinical context, easy to use, and accompanied by appropriate training and on-going support. Research to address these needs and establish evidence to support the national adoption of standard diagnostic methods is required to improve the diagnostic capacity for FASD in Australia. The importance of establishing an evidence base for diagnostic and management practices is further reinforced by research which indicates that FAS may not be diagnosed if service providers do not believe that it will make a difference to the individual [25].

The perceived limitations of existing diagnostic guidelines that were identified by participants in this study correspond with limitations identified in the literature, and many represent current challenges to the effective diagnosis of individuals with FASD [1,11]. Although the UW guidelines were recognised as having been developed based on empirical data, they were not always considered practical for the clinical setting. In contrast, the Canadian guidelines, which integrate elements of the UW and IOM approaches to diagnosis, and were developed based on published literature, expert opinion and best practices [7], were not always considered evidence based.

As acknowledged in the Canadian guidelines, a lack of evidence in key areas, including growth reference standards for all cultural groups, and of screening tools specific and sensitive to prenatal alcohol exposure, limits the effectiveness of the current diagnostic process [7]. Canadian palpebral fissure length growth charts [26], which have recently been found to be applicable to children in the United States [27], are likely to be suitable for use in Australia; however, their appropriateness requires formal evaluation. Australian standards for Indigenous Australians also need to be developed. The lack of evidence can also be problematic where limitations of current diagnostic methods, such as the absence of identified biological markers of alcohol exposure during pregnancy, preclude the diagnosis of neurodevelopmental disorders where there is no confirmed evidence of prenatal alcohol exposure, despite information suggesting that alcohol exposure is likely. Diagnostic methods for FASD will be facilitated by improvements in medical technology and increased understanding of the disorders [16].

The UW and Canadian guidelines are similar, with both covering a spectrum of diagnostic outcomes, excluding the diagnostic category of alcohol-related birth defects, and including almost identical diagnostic criteria [16]. As highlighted by participants, these two guidelines also have important differences, including the diagnostic terminology used and the definition of central nervous system abnormalities. The terminology used in the UW guidelines was both perceived as being too complex, and preferred for eliminating the implication that alcohol is the primary causative factor for these disorders. The criteria for central nervous system abnormality included in the Canadian guidelines were also perceived as restrictive due to their reliance on testing for central nervous system dysfunction, which may not be possible in young children and therefore may decrease the likelihood of early diagnosis.

Perceptions of the strengths and limitations of diagnostic guidelines may conflict, and are likely to reflect: i) variation in the nature of the guidelines examined, including their evidence base and method of development; and ii) variation in individual characteristics, beliefs, experience and knowledge, including the context of experience, and extent of familiarity with diagnostic guidelines for FASD. In this study the perceived limitations of existing guidelines were evaluated in the context of their use in Australia, and our findings may not reflect their perceived limitations in other contexts. The ability of participants to make more general comparative evaluations among guidelines was also constrained by the small proportion of participants who were familiar with more than one guideline. As a result, perceived limitations reported for one guideline may also apply to other guidelines.

Despite the range of factors that influence individual perceptions of guidelines and the inability of many participants to evaluate multiple guidelines, an understanding of perceptions about existing guidelines enables consideration of factors influencing the utility and adoption of existing guidelines in Australia. Reported issues and identified needs may be addressed through guideline design and implementation, training, research, and health policy. Although the reported limitations provide valuable information, not all can be addressed in the short term. Some perceptions highlight current challenges to diagnosis irrespective of the guideline used, and reinforce appreciation of the need for improved technologies for diagnosis. Other perceptions highlight policy issues relevant to FASD diagnosis in Australia that need to be addressed. These include the lack of sufficient resources to enable access to interdisciplinary diagnostic assessment in Australia despite clear consensus in the literature on the need for interdisciplinary team assessment for FASD diagnosis [4-7], the need for a standard national approach to diagnosis, and the lack of evidence base to support accurate and appropriate diagnosis, surveillance and management in the Australian context.

The validity of our findings is supported by the evidence of association found between individual characteristics and familiarity with and use of existing diagnostic guidelines. Health professionals who had not completed specific training in diagnosis had poorer knowledge of diagnostic guidelines for FASD. This finding is consistent with previous work indicating that many paediatricians identified their need for educational materials on diagnosis [24]. Participants who reported experience in diagnosis and completion of specific training in FASD diagnosis were also more likely to report having used the UW guidelines, consistent with both the prominent use of the UW guidelines internationally [12] and the accessibility of comprehensive online training in the UW diagnostic methods [28], which we found to be a factor influencing the use of a specific guideline. These findings also highlight areas where policy and education could strengthen diagnostic capacity for FASD.

This survey was conducted within a larger study of health professionals’ perceptions of FASD diagnosis in Australia, and the length of the questionnaire may have discouraged participation among some individuals. We purposively sampled participants to identify health professionals who had expertise in or experience with the screening or diagnosis of FASD and could provide information relevant to the adoption of national diagnostic guidelines. As such, our findings are not generalisable to all health professionals in Australia. A small sample of international participants with recognised expertise in FASD was also recruited, and the differences demonstrated in responses between Australian and international participants reflect both the nature of the sampling process and the lack of progress on the implementation of standard diagnostic guidelines for FASD in Australia.

Despite the targeted recruitment of health professionals in this study, our findings were limited by the small number of participants who had used existing diagnostic guidelines and could evaluate their suitability in the Australian context. Participant perceptions of the limitations of existing systems may also vary based on a range of factors, including the clinical context in which the guidelines are used. Nevertheless, the participating health professionals indicated support for a national guideline, provided valuable information about the suitability of existing guidelines for the Australian context, and identified factors which may influence adoption and help ensure the appropriateness of national recommendations for diagnosis. This survey was conducted to enable evidence from a national consultation and consensus development process with health professionals to be considered in the design of an Australian diagnostic instrument for FASD. The national approach to development will utilise findings from this survey, findings from the broader consultation process, and evidence from the published literature.

## Conclusions

Our findings demonstrate a lack of certainty among health professionals about the most appropriate guideline for use in Australia, and some support for the adoption of the UW or Canadian guidelines. Nevertheless, health professionals endorsed the need for national diagnostic guidelines for FASD, and the need for their evaluation in the Australian context to ensure that the guidelines are feasible, nationally applicable, valid, and acceptable to both health professionals and consumers. Health professionals support a nationally coordinated approach to the diagnosis of FASD. This should incorporate standard diagnostic criteria, include an interdisciplinary and interagency model of service delivery, and address health professionals’ concerns about existing diagnostic criteria. Training and support for service providers in the use of the diagnostic guidelines would be necessary to increase diagnostic capacity in Australia.

## Competing interests

The authors declare that they have no competing interests.

## Authors’ contributions

CB, EJE and JMP designed the study and CB and EJE supervised the study. CB, EJE, REW, JL and HJ designed the study questionnaire, and all authors were members of a project steering group that reviewed the study methods and procedures. REW programmed the online questionnaire and analysed the data. REW and CB drafted the manuscript and all authors critically reviewed the manuscript. All authors read and approved the final version of the manuscript.

## Pre-publication history

The pre-publication history for this paper can be accessed here:

http://www.biomedcentral.com/1471-2431/12/69/prepub
